# Iron Forms Fe(II) and Fe(III) Determination in Pre-Roman Iron Age Archaeological Pottery as a New Tool in Archaeometry

**DOI:** 10.3390/molecules26185617

**Published:** 2021-09-16

**Authors:** Lidia Kozak, Andrzej Michałowski, Jedrzej Proch, Michal Krueger, Octavian Munteanu, Przemyslaw Niedzielski

**Affiliations:** 1 Department of Analytical Chemistry, Faculty of Chemistry, Adam Mickiewicz University in Poznań, 8 Uniwersytetu Poznanskiego Street, 61-614 Poznań, Poland; lkozak@amu.edu.pl (L.K.); jedrzej.proch@amu.edu.pl (J.P.); 2 Faculty of Archaeology, Adam Mickiewicz University in Poznań, 7 Uniwersytetu Poznanskiego Street, 61-614 Poznań, Poland; misiek@amu.edu.pl (A.M.); krueger@amu.edu.pl (M.K.); 3 Interdisciplinary Research Group Archaeometry, Faculty of Archaeology and Faculty of Chemistry, Adam Mickiewicz University in Poznań, 7–8 Uniwersytetu Poznanskiego Street, 61-614 Poznań, Poland; 4 World History Department, State Pedagogical University, 1 Ion Creanga Street, MD-2069 Chisinau, Moldova; ocmunteanu@gmail.com

**Keywords:** pottery, iron, speciation, archaeometry, spectrophotometry, spectrometry, pre-Roman Iron Age

## Abstract

This article presents studies on iron speciation in the pottery obtained from archaeological sites. The determination of iron forms Fe(II) and Fe(III) has been provided by a very simple test that is available for routine analysis involving the technique of molecular absorption spectrophotometry (UV–Vis) in the acid leachable fraction of pottery. The elemental composition of the acid leachable fraction has been determined by inductively coupled plasma optical emission spectrometry (ICP-OES). Additionally, the total concentration of the selected elements has been determined by X-ray fluorescence spectrometry with energy dispersion (EDXRF). The results of the iron forms’ determinations in archaeological pottery samples have been applied in the archaeometric studies on the potential recognition of the pottery production technology, definitely going beyond the traditional analysis of the pottery colour.

## 1. Introduction

Clay is a natural rock material that has been used as a building material by people for over 10,000 years due to its availability and properties [1]. From a chemical perspective, it combines one or more silicate minerals, also known as phyllosilicates, as well as traces of metals, metal oxides, sand, and organic matter. Clay minerals are usually hydrated, and they exhibit plasticity due to their water content. After drying and/or firing, they lose their water content, and clay itself becomes hard and non-plastic [2]. Crafting vessels from clay was definitely a large step in the development of prehistoric communities. Ceramics and their properties, such as quality, element or water content, or even shapes and patterns, are very important indicators in archaeometric research that can lead us to various conclusions about the past [3].

Archaeometry is an important branch of archaeology that applies various scientific techniques in order to acquire more information about archaeological samples, such as pottery [4], metal artefacts, [5] or building materials [6,7]. The archaeometric study of ceramics is especially important because the pottery was used daily by settled farming communities [8]. Applying scientific methods to ubiquitous ceramic samples found at various excavation sites helps us to find answers about the date and place of the pottery production, the likely technique and parameters used during firing, or the purpose of their creation [9,10,11]. Admixtures (and also clay firing parameters) can be markers providing information about connections between samples found in different places [12]. Redox and phase speciation (for solid samples) can be used to determine the parameters in which the studied object was formed [13]. Moreover, it can be used to understand the processes that changed the chemical composition of an object from the state when it was formed [14]. Ceramics as works of art are often painted on the surface. There are numerous examples of element speciation studies in ceramics in which the oxidation state of the elements was the subject of determination: iron and manganese in Sicilian “proto-majolica” pottery [15], iron in black glaze of Chinese pottery [16], cobalt in Chinese porcelains from the 16th to 17th century [17], and iron in ceramics from Brazil [18].

The pigments used in coatings or glazes are often metal oxides in different oxidation states, which differ in colour [13]. Moreover, different parameters during clay firing are responsible for elements’ oxidation state distribution, which is visible as a change in the appearance of the ceramic form. Iron is an element that is the component of many silicate minerals, such as illite, chlorite, glauconite, or biotite [19]. The determination of iron speciation in pottery is especially important because various types of clay, just like different clay firing techniques and temperatures, have a major influence on the oxidation state and—furthermore—on the colour of ceramics [18]. Fe(III) is responsible for an orange-red colour, whereas Fe(II) imparts a dark grey colour. In practice, both forms can simultaneously exist in ceramics and their coatings, so the determination of the Fe(III)/Fe(II) ratio is of great importance (Orecchio, 2011). The simplest way to quantitatively determine Fe(II) and Fe(III), is to carry out a mineralisation of a powdered sample by hydrofluoric acid and to use UV–Vis spectrophotometric methods in order to determine Fe(II) and Fe(III). A similar method was proposed for studies of iron speciation in ancient pottery from Sicily [20]. The sample was mineralised, and then the method for determining Fe(II) by the reaction of Fe^2+^ with 1,10-phenanthroline was applied [21]. Moreover, the author determined the total iron using ICP-OES, and the results were in agreement with those of the phenanthroline method [20]. There are many other analytical methods for determining iron speciation, but they require more advanced equipment. One of them uses Mössbauer spectroscopy, which is based on the Mössbauer effect. It was applied in the recognition of the oxidation state of iron in Brazilian ceramic samples, along with Scanning Electron Microscope (SEM), X-ray fluorescence (XRF), and X-ray diffraction (XRD) [18].

A non-destructive X-ray absorption spectroscopy (XAS) is also used for archaeological artifacts analysis. Compared with XRD, it does not require ordered structures [22]. Therefore, it can be applied to amorphous samples with no restrictions on their type or size [23]. In addition, the Extended X-ray Absorption Fine Structure (EXAFS) range shows the local structure around the iron sites. The whole study demonstrates numerous possibilities for analysing samples by XAS [20].

In this article, the studies on iron speciation in archaeological pottery are described. The results of the determination of iron forms: Fe(II), Fe(III) and chemical composition (the occurrence of the selected elements, both total concentration and occurrence in the acid leachable fraction of pottery) are compared for different archaeological sites.

## 2. Experimental

### 2.1. Instrumentation

For determination of the total concentration of selected elements (As, Ba, Ca, Co, Cr, Cu, Fe, Mn, Mo, Nb, Ni, Pb, Rb, Sb, Sn, Sr, Th, Ti, U, Y, Zn, Zr) in pottery samples, the portable XRF spectrometer Tracer III Handheld XRF Bruker (Billerica, MA, USA) was used. The spectrometer has worked in quantitate mode (determination limits on the level of 1 mg kg^−1^, uncertainty level below 15%) elaborated by the manufacturer for geochemical analysis with two built-in calibrations: Bruker Mudrock Major (Al, Ba, Ca, Fe, K, Mg, Mn, and Ti determination; instrumental parameters: 15 keV, 25, μA, vacuum < 17 Torr); and Bruker Mudrock Trace (As, Co, Cr, Cu, Mo, Nb, Ni, Pb, Rb, Sb, Sn, Sr, Th, U, Y, Zn, Zr determination; instrumental parameters: filter 0.3048 mm Al and 0.0254 mm Ti, 40 kV, 12 µA). The problem of accuracy in studies was discussed in the Supplementary Data.

ICP-OES spectrometer Agilent 5110 ICP-OES Agilent (Santa Clara, CA, USA) was used in selected elements (Al, As, B, Ba, Bi, Ca, Cd, Ce, Co, Cr, Cu, Dy, Er, Eu, Fe, Ga, Gd, Ge, Ho, In, K, La, Li, Lu, Mg, Mn, Mo, Na, Nd, Ni, Pb, Pd, Pr, Re, Rh, Sb, Sc, Se, Sm, Sr, Tl, Tm, Y, Yb, Zn) for determination in simultaneous mode. The simultaneous axial and radial view of plasma was allowed by the synchronous vertical dual view (SVDV). For multi-elemental determination, the common conditions were used: 3 replicates, measuring time 5 s, plasma gas flow 12.0 L min^−1^, auxiliary gas flow 1.0 L min^−1^, nebulizer gas flow 0.7 L min^−1^, Radio Frequency (RF) power 1.2 kW. For determination of higher level of the selected elements, the alternative (less sensitive) wavelengths were used (indicated by bold in Table S1). Spectrometer build-in method of background correction (fitted) was used. The detection limits were calculated based on the standard deviation value of multiple (*n* = 10) calibration blank analysis: 3-sigma criteria. The detection limits for all determined elements were in the range of 0.01–0.09 mg kg^−1^ (Table S1 in Supplementary Data).

The uncertainty budget was estimated for the complete analytical procedure, including preparation of samples, instrument calibration, and determination of the content of elements. The propagated uncertainty (a coverage factor k = 2 for approximate 95% confidence) was at a level below 20%.

Due to the lack of access to standard reference materials (CRM) for the multi-elemental pottery analysis, the soil and sediments matrix CRM was used because of its geological similarity to the raw materials of ceramics (post-glacial and sedimentary materials). For the traceability studies, the following CRMs were selected: NIST 2709—soil; IAEA 405—estuarine sediments; CRM S-1—loess soil; BCR 667- estuarine sediments (Table S2 in Supplementary Data). Due to the fact that the information about elements’ concentration of acid extractable fraction was available only for NIST 2709a, the analysis of CRMs were provided using two procedures: (i) with sample digestion using the mixture of concentrated HCl and HNO_3_ (aqua regia (AR)); (ii) with sample extraction by HCl following the procedure described below. The first step allowed the calibration and interferences correction to be checked (using spectrometer build-in background correction method); the second step allowed the matrix-dependent interferences to be controlled. Additionally, the standard addition method was applied for HCl extracts. In all procedures, the acceptable recovery (in the range 80–120%) was found (Table S3 in Supplementary Data). In colorimetric analyses, the photometer Slandi LF300 Slandi (Michalowice, Poland) was used (measurements of absorbance at 470 and 520 nm). The detection limits for both iron forms were determined by dilution of calibration standards, and the levels of 10 mg kg^−1^ were determined for Fe(II) and Fe(III), respectively, with uncertainty below 15%. To check the accuracy, the standard addition method was applied with good recovery (in the range 80–120%, Table S4 in Supplementary Data). Additionally, the reference procedures were applied in accuracy studies (Table S5 and procedures description in Supplementary Data).

For homogenisation of the samples, the Pulverisette agate laboratory grinder Fritsch (Idar-Oberstein, Germany) was used.

### 2.2. Reagents

Only analytical purity reagents and deionised water from a Milli-Q device Millipore (Burlington, VT, USA) were used. Standard solutions (1.00 g L^−1^) of iron forms: Fe(III) and Fe(II) were prepared from ferric ammonium sulphate dodecahydrate and ferrous ammonium sulphate hexahydrate Acros-Thermo Fisher Scientific (Geel, Belgium), respectively. The commercial standards (1.000 g L^−1^) were used for ICP-OES analysis Romil (Cambridge, UK). The following reagents POCh (Gliwice, Poland) were used: 2.0 mol L^−1^ solution of hydrochloric acid (HCl), 0.5% (m/m) solution of 2,2′-Bipirydyl (C_10_H_8_N_2_), acetate buffer (sodium acetate and acetic acid), 5% (m/m) solution of potassium thiocyanate (KSCN).

### 2.3. Samples

The 78 fragments of pottery chosen for chemical analysis came from three archaeological sites from western Poland. Sites were located in close proximity to each other: Borzejewo (B) and Poznań-Nowe Miasto (P), located in central Wielkopolska, and Grabkowo (G) in the eastern part of Kujawy. The reference material for the mentioned sites was the archaeological site of Poieneşti-Lukaševka Culture from Orcheiul Vechi (M) from Moldova. The whole collection of analysed samples can be combined with a unified time horizon—associated with the early phases of the younger pre-Roman Iron Age and an approaching cultural component visible in the material, initially referred to as Jastorf Culture. This also applies to the areas of Moldova. The collection of this reference material was the result of an attempt to capture potentially similar cultural factors that could be manifested in radically different raw materials. Furthermore, 126 pottery fragments from Pławce (Pl) in western Poland were selected for comparative analysis. In the case of this latter site, the selection criteria were the area of origin and cultural phenomena readable in archaeological material analogous to other previously mentioned sites. An important factor was to implement an analysis of all samples taken from the archaeological site.

### 2.4. Methodology

The acid leaching procedure was described for geochemical studies [24]. The established analytical procedure of the speciation analysis [25,26] was optimised and applied for pottery samples. The analysis by XRF technique was described in previous work [27]. The sample preparation procedure is identical to the one applied for metals determination described in previous work [28]. The accuracy of the studies regarding all of the analytical procedures has been described in the Supplementary Data.

### 2.5. Total Iron and Selected Elements Determination

The XRF analysis was provided in laboratory using desktop spectrometer-stand. The pottery sample was placed on the spectrometer stand, oriented in correspondence with the original external surface of the ceramic vessel, and analysed. After analysis, the sample was rotated and the analysis was repeated. The acquisition time was 15 s. The mean value of concentration of selected elements (As, Ba, Ca, Co, Cr, Cu, Fe, Mn, Mo, Nb, Ni, Pb, Rb, Sb, Sn, Sr, Th, Ti, U, Y, Zn, Zr) and relative standard deviation were calculated from three repetitions (*n* = 3).

### 2.6. Acid Leachable Fraction Analysis

#### 2.6.1. Sample Extraction by Hydrochloric Acid

The extraction by hydrochloric acid (methodology of acid leaching) was prepared following the previous studies [24]. The ceramic material was homogenised by grinding; the coarse material was removed using a plastic sieve (diameter of particle > 0.02 mm). Samples weighed to be 2.00 ± 0.01 g were put into a flask and 20 mL of hydrochloric acid solution (2 mol L^−1^) was added. The flask (with a reflux condenser) was heated to approximately 80 °C for 30 min. After cooling, the solution was drained through a paper filter (rinsed previously using 200 mL of water) into a test tube; finally, water was added to a volume of 50.0 mL.

#### 2.6.2. Elemental Analysis

Hydrochloric acid extracts of samples were analysed using ICP-OES technique. The selected elements were determined (indicated in the Supplementary Data).

#### 2.6.3. Iron Chemical Forms Determination

The content of Fe(III) was determined using reaction of Fe(III) with thiocyanate in pH < 2.0 (in the hydrochloric acid environment). The intensity of light absorption (absorbance) by red complex was measured at wavelength 470 nm by UV–Vis spectrophotometer and compared with the calibration curve prepared using Fe(III) standard. The content of Fe(II) was determined in reaction of Fe(II) with 2,2′-bipirydyl (in the acetate buffer (pH 4.5). The intensity of light absorption (absorbance) by red complex was measured at wavelength 520 nm by UV–Vis spectrophotometer and compared with the calibration curve prepared using Fe(II) standard.

### 2.7. Statistical Analysis

The analysis of the experimental data was performed using computer software Statistica 13.1 StatSoft—Dell (Round Rock, TX, USA). The multidimensional statistical analysis (principal components analysis PCA) was provided for the results of XRF and ICP-OES analysis to indicate the individual differences in the elemental composition of the pottery samples. For all statistical tests, the probability value *p* = 0.05 was applied [29,30].

## 3. Results and Discussion

### 3.1. Analysis of the Pottery Fragments from Four Sites

#### 3.1.1. Chemical Composition of Pottery

**Figure 1:**
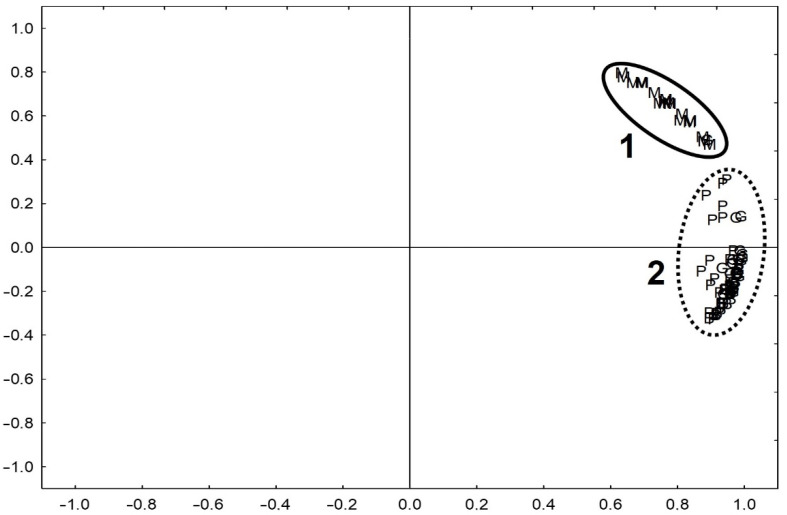
Results of Principal Components Analysis for elements concentration in pottery samples from four archaeological sites (Borzejewo (B), Poznań-Nowe Miasto (P), Grabkowo (G), Moldova(M)); 1—the group of samples from site M (Moldova), 2—the group of samples from the other sites.

The results (*n* = 4964 of single results for 78 fragments of pottery from sites B, G, P and M) of the total concentration of the elements (obtained in the XRF analysis of the raw material) and the concentration of elements in the acid leachable fraction (obtained in the ICP-OES analysis of the HCl extracts) were analysed using exploratory analysis (PCA). The 95.5% variability of the results was described by two components (Figure 1).

It is clearly indicated that the elemental composition of the studied samples is different for the pottery from Moldova and Poland. The difference is based on the geology of the regions: the Moldovan clays were marine deposits, while, in Poland, the formation of the clays was connected with the glacial and post-glacial processes. Different raw materials (clay) were used for the production of the pottery, and they were indicated in the composition of the material.

#### 3.1.2. Iron Speciation in Pottery

**Figure 2:**
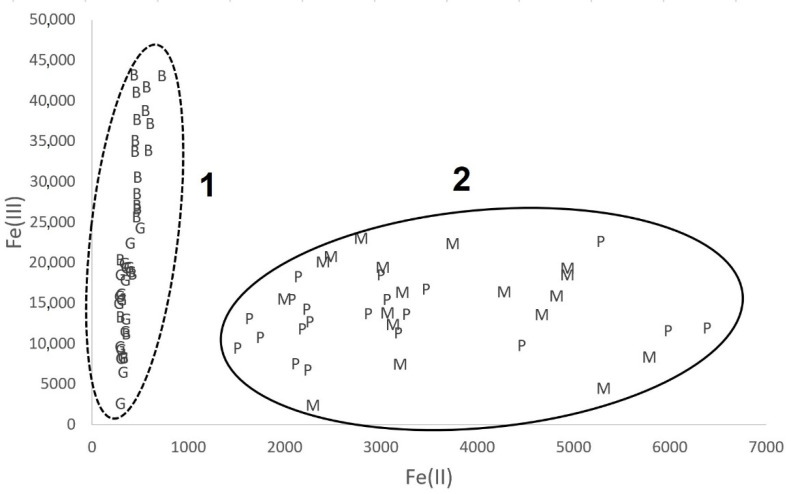
The iron speciation in pottery samples: 1—the group of samples from sites Borzejewo (B) and Grabkowo (G); 2—the group of samples from sites Poznań-Nowe Miasto (P) and Moldova(M).

The results of the determination of iron forms (Fe(II) and Fe(III)) were put together in Figure 2. Two groups of pottery samples were formed: the first for pottery from archaeological sites in Bozejewo (B) and Grabkowo (G)—group 1 in Figure 2—and the second one for pottery from Poznan (P) and Moldavia (M)—group 2.

**Figure 3:**
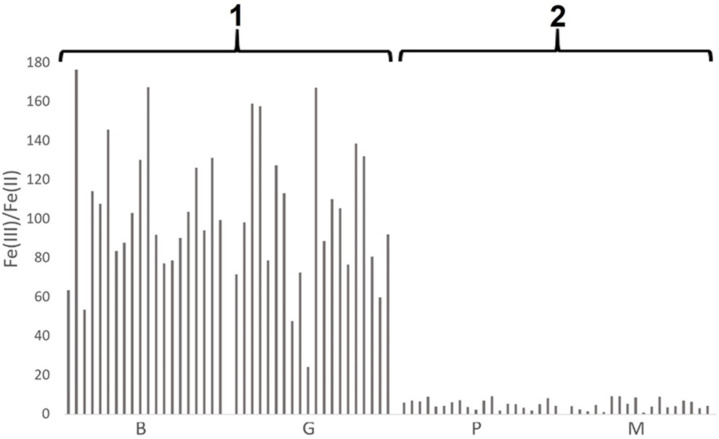
The Fe(III)/Fe(II) ratio for samples from sites Borzejewo (B), Poznań-Nowe Miasto (P), Grabkowo (G), Moldova(M); 1—the group of samples with Fe(III)/Fe(II) ratio greater than 20 (sites (Borzejewo (B) and Grabkowo (G))); 2—the group of samples with Fe(III)/Fe(II) ratio less than 20 (sites (Poznań-Nowe Miasto (P) and Moldova(M))).

The similarities of the iron speciation are different to the shapes of the chemical compositions of the samples. Due to the different origin of the raw material (marine clay versus glacial or post-glacial clay) used for pottery production, the pottery chemical composition of the samples from the Poznan archaeological site is different to the pottery chemical composition of the samples from Moldavia. The differences in the material from which the ceramics are made are not reflected in the iron speciation. According to the literature data, the presence of Fe (III) and Fe (II) forms and their Fe(III)/Fe(II) ratio reflect the technology of the ceramics production process [18], particularly the temperature and conditions of the ceramics firing process [31]. Thus, it can be concluded that, regardless of the origin of the ceramics tested, and, therefore, the material (clay) from which it was made, two groups of ceramic objects (Figure 3) stand out on the basis of iron speciation, probably as a result of similar technological processes.

The first group consists of ceramics from two archaeological sites (B and G), characterized by high values of the Fe(III)/Fe(II) ratio, exceeding the range from 24 to 176 (median values 101 and 95 for site B and site G, respectively). The predominant form of iron was that of Fe(III), which indicates both a higher ceramic firing temperature and the provision of an oxidizing environment during firing [20]. On the other hand, for the second group of samples (P and M), the value of the ratio Fe(III)/Fe(II) was definitively lower, ranging between 1 and 9 (median values 5 and 4 for site P and site M, respectively). This indicates a definitively higher concentration of Fe(II) than for the first group, which indicates technology using a lower ceramic firing temperature, with a definitively limited supply of oxidizing agent [32]. Importantly, the ceramic firing techniques, although characteristic of a given archaeological site, were unrelated to both the origin of the source material (clay) and the location of the site.

### 3.2. Analysis of Pottery Fragments from One Site

In the second step of the research, the mass pottery material obtained from one archaeological site (Plawce—Pl) was analysed. The 126 pottery samples represented all of the samples collected at the given site without any pre-selection or elimination. This creates the opportunity to compare fragments of ceramics made of a similar material but resulting from the actions of various pottery manufacturers.

#### 3.2.1. Chemical Composition of Pottery

**Figure 4:**
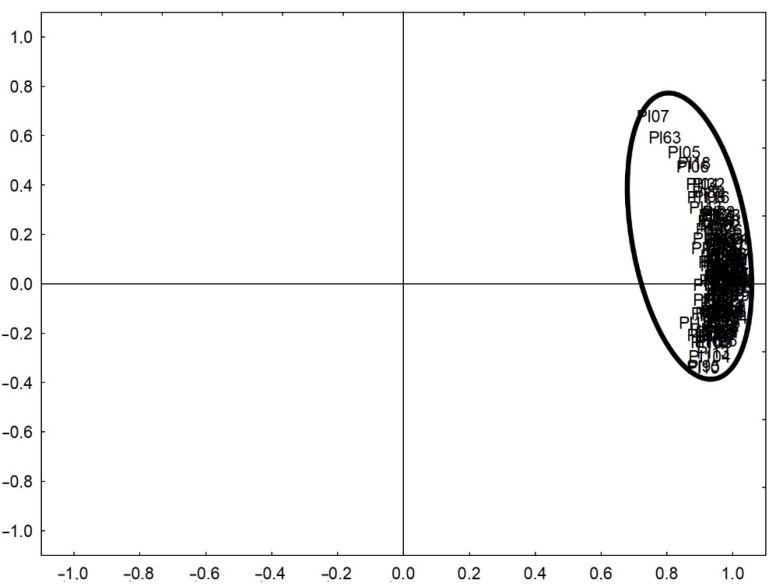
Results of Principal Components Analysis for elements concentration in pottery samples from Pl archaeological site.

The results (*n* = 9198 of single results for 126 fragments of pottery from sites Pl) of the total concentration of the elements (obtained in the XRF analysis of the raw material) and the concentration of elements in the acid leachable fraction (obtained in the ICP-OES analysis of the HCl extracts) were analysed using exploratory analysis (PCA). The 96.0% variability of the results was described by two components (Figure 4).

The results of the exploratory analysis indicated that the actually tested ceramics were characterized by a very similar chemical composition, which allows the hypothesis of the local exploitation of one source of clay by ceramic manufacturers at the time to be considered. Thus, we were dealing with a homogeneous set of ceramic fragments in terms of chemical properties.

#### 3.2.2. Iron Speciation in Pottery

**Figure 5:**
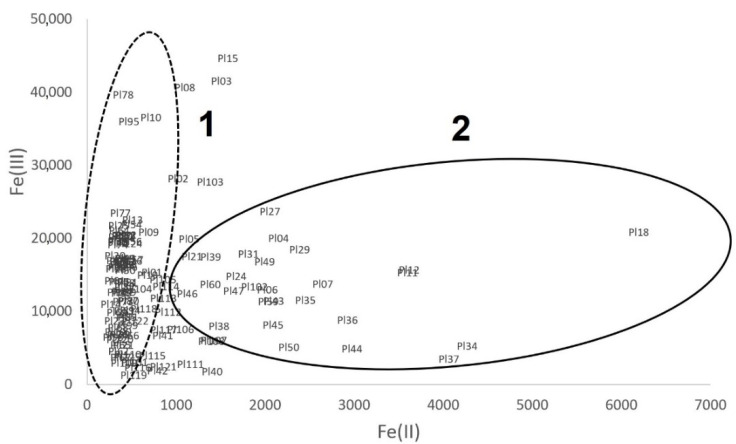
The iron speciation in pottery samples: 1—the group of samples “similar to” pottery from sites B and G; 2—the group of samples “similar to” pottery from sites M and P.

The results of the determinations of iron forms (Fe(II) and Fe(III)) were presented together in Figure 5. Two groups of pottery samples were indicated based on Figure 2: the first for pottery similar to pottery from archaeological sites in Bozejewo (B) and Grabkowo (G)—group 1 in Figure 5, the second one for pottery “similar to” pottery from Poznan (P) and Moldavia (M)—group 2.

**Figure 6:**
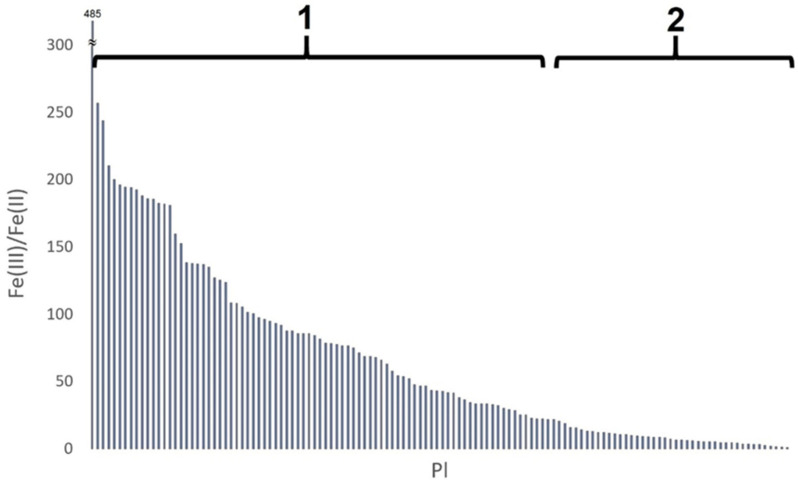
The Fe(III)/Fe(II) ratio for samples from site Pl: 1—the group of samples with Fe(III)/Fe(II) ratio greater than or equal to 20; 2—the group of samples with Fe(III)/Fe(II) ratio less than 20.

The factor Fe(III)/Fe(II) for samples from site Pl was shaped in a very broad range: from 1 to 485 (Figure 6).

For most (85) samples, the value of the Fe(III)/Fe(II) ratio was greater than or equal to 20. As stated above [20], this indicated a higher ceramic firing temperature, as well as the presence of an oxidizing atmosphere during firing. For a smaller number of samples (41), the value of the Fe(III)/Fe(II) ratio was less than 20, indicating different firing conditions: a lower temperature and a reducing atmosphere [32].

Despite the similar chemical composition of the examined ceramic fragments, suggesting the origin of the raw materials from the same or a very geologically similar source, the iron speciation showed a wide variation within the considered sample collection. The reason for this could be the different technology used in different pottery workshops operating in the same place. In addition, a large variation in the value of the Fe(III)/Fe(II) ratio may indicate the variability of the firing conditions even within one workshop. The high variability of the results for the Fe(III)/Fe(II) coefficient may arise for two reasons: (i) the inhomogeneity of the distribution of iron forms in single vessels; (ii) the high variability of the firing conditions even within one pottery workshop. However, the inhomogeneity indicated in point (i) may also result from the instability of the firing process and the lack of its full repeatability. Although the group of analyzed pottery fragments was not large, the results allow for the formulation of a hypothesis about the large differentiation and randomness of the conditions of ceramics firing within one site.

## 4. Conclusions

The speciation analysis has been demonstrated to be a useful tool for archaeometrical studies that make it possible to obtain new information about the characteristics of pottery. However, the speciation studies require the destruction of a part of a sample (although this is not problematic for mass pottery materials). The very simple, well-known, and, for most researchers, readily available analytical procedures enable analysis of a great number of samples. A large dataset is the ideal condition to provide a statistical analysis that can be used to find the similarities and differences in the pottery as it pertains to the results of, e.g., pottery manufacturing. It was found that the values of the Fe(III)/Fe(II) coefficient do not depend on the origin of the original material from which the ceramics were made. On the one hand, similar values of the Fe(III)/Fe(II) coefficient were obtained for pottery from regions with different geology (clays from marine deposits versus clays whose formation is connected with the glacial and post-glacial processes). On the other hand, the coefficient values were different for pottery samples with a similar chemical composition coming from the same area. Based on the literature, the reason for the differentiation of the Fe(III)/Fe(II) coefficient can be indicated by the different technological processes used in the manufacture of ceramics.

The most important novelty of the work is the development of simple, routine analytical tools that enable the indication of the technology of producing ceramics. The possibility of applying the developed procedures in the analysis of a large number of samples allows the use of statistical analysis to interpret the results, which is not possible with the traditional classification of ceramics based on their colour.

## Data Availability

All data presented in the article are available from the corresponding author.

## Supplementary Materials

The following are available online. Description of XRF accuracy studies, ICP-OES analytical figures of merit: Table S1. Wavelengths used in analysis, detection limits (DL), calibration range (range) and precision for ICP-OES determination, ICP-OES accuracy studies: Table S2. Results of certified reference materials analysis, Table S3. Results of acid extractable fraction of certified reference material NIST 2709a analysis and spike recovery in standard addition method, Colorimetric analysis accuracy studies: Table S4. Spike recovery in colorimetric procedures, Table S5. Results of iron forms determination using different colorimetric procedures and hyphenated analytical systems References to previous works.
