# Supplementary material for: Iron Forms Fe(II) and Fe(III) Determination in Pre-Roman Iron Age Archaeological Pottery as a New Tool in Archaeometry
Source: Molecules. 2021 Sep 16;26(18):5617. doi: 10.3390/molecules26185617 (PMC8472336; doi:10.3390/molecules26185617)
Supplement: Supplementary file 1 [file molecules-26-05617-s001.zip › molecules-1334002-supplementary.pdf]

Supplementary data

Iron forms Fe(II) and Fe(III) determination in Pre-Roman Iron Age archaeological pottery as a new tool in archaeometry

Lidia Kozak, Andrzej Michałowski, Jedrzej Proch, Michal Krueger, Octavian Munteanu, Przemyslaw Niedzielski

XRF accuracy studies

The most problematic issue is the use of independent analytical technique as reference method in analysis of pottery from archaeological sites. XRF allows to use a non-destructive technique to analyze pottery fragments while most of the spectrometric techniques requires grinding the sample and then its decomposition or extraction of the determined components. In previous work [S1] the effects of sample preparation were observed on results of XRF measurements. The results obtained for the analysis of pottery samples and the same melted samples cannot be compared. Non-destructive analysis and analysis of powdered samples, even with the same instrument, are two completely different analytical procedures. Therefore, both types of analyses cannot be treated interchangeably, but may complement each other and extend the scope of interpretation.

In view of the problems described above, the use of the XRF technique (using a different spectrometer) as the reference method remains. The studies conducted for postglacial sediments [S2] (the primary raw material for the production of ceramics) indicated that the results of analyses performed with handheld XRF spectrometers from different manufacturers differ statistically. However, despite the differences in the results obtained with different spectrometers, they reflect the spatial distribution of elements in the studied area, making XRF a useful tool in geochemical research.

Therefore, the control of results accuracy is problematic and has been recognised in our previous work [S3]. In determining the accuracy of the obtained results, an assumption should be made about the homogeneity of the analysed samples and a similar nature of the analysis (e.g. spectral or matrix-dependent interferences) of all the elements determined. In the validation process, the results obtained in the analyses of selected samples should be representative for the remainder of the research material. The above assumption can therefore be applied for statistical analyses. In the case of exploratory statistical analysis, the accuracy of the results of chemical analyses is not important, because the statistical tools are not the elemental composition of the selected sample, but the variability of the level of all the studied quirks. Thus, even when the accuracy of the XRF analyses is difficult to prove, a statistical analysis can be successfully performed.

ICP-OES analytical figures of merit

Table S1. Wavelengths used in analysis, detection limits (DL), calibration range (range) and precision for ICP-OES determination

	**wavelength**	**DL**	**range**	**precision**		**wavelength**	**DL**	**range**	**precision**
	nm	mg kg^{-1}	mg kg^{-1}	%		nm	mg kg^{-1}	mg kg^{-1}	%
**Al**	**394.401**	**nd**	**200-4000**	**6.5**	Li	670.783	0.0044	DL-200	16.6
Al	396.152	0.0053	DL-200	2.9	Lu	307.760	0.031	DL-100	2.9
As	188.980	0.012	DL-100	14.6	Mg	279.553	0.008	DL-500	9.7
B	249.772	0.047	DL-200	3.4	**Mg**	**285.213**	**nd**	**500-5000**	**4.5**
Ba	455.403	0.002	DL-200	9.0	Mn	257.610	0.0021	DL-200	1.8
Bi	223.061	0.065	DL-200	4.1	Mo	202.032	0.044	DL-100	3.1
**Ca**	**315.887**	**nd**	**2000-10000**	**6.4**	Na	588.995	0.26	DL-1000	6.7
Ca	422.673	0.036	DL-2000	2.8	**Na**	**589.592**	**nd**	**1000-10000**	**3.4**
Cd	214.439	0.0026	DL-200	10.1	Nd	406.108	0.012	DL-100	6.2
Ce	446.021	0.023	DL-100	13.6	Ni	231.604	0.0092	DL-200	8.0
Co	238.892	0.0029	DL-200	1.6	Pb	220.353	0.023	DL-200	4.2
Cr	267.716	0.0033	DL-200	4.0	Pd	340.458	0.025	DL-100	9.0
Cu	327.395	0.0027	DL-200	9.6	Pr	417.939	0.032	DL-100	9.8
Dy	364.540	0.023	DL-100	6.7	Re	197.248	0.032	DL-100	5.0
Er	349.910	0.018	DL-100	7.9	Rh	343.488	0.035	DL-100	13.3
Eu	420.504	0.034	DL-100	3.0	Sb	206.834	0.012	DL-100	14.0
Fe	238.204	0.0084	DL-1000	1.6	Sc	361.383	0.024	DL-100	3.0
**Fe**	**261.382**	**nd**	**100-4000**	**1.0**	Se	196.026	0.011	DL-100	16.4
Ga	294.363	0.0097	DL-200	3.4	Sm	442.434	0.026	DL-100	9.8
Gd	342.246	0.034	DL-100	6.1	Sr	460.733	0.0092	DL-200	5.3
Ge	209.426	0.039	DL-100	2.2	Tl	190.794	0.024	DL-200	7.3
Ho	348.484	0.031	DL-100	11.9	Tm	336.261	0.031	DL-100	7.6
In	230.606	0.025	DL-200	8.0	Y	361.104	0.031	DL-100	6.2
K	766.491	0.34	DL-1000	1.3	Yb	328.937	0.014	DL-100	3.8
**K**	**769.897**	**nd**	**1000-10000**	**1.0**	Zn	213.857	0.0022	DL-200	3.5
La	398.852	0.0086	DL-100	6.8					

nd – detection limit not determined

ICP-OES accuracy studies

Table S2. Results of certified reference materials analysis

	NIST 2709a			IAEA 405			CRM S-1			BCR 667		
	certified	determined	recovery	certified	determined	recovery	certified	determined	recovery	certified	determined	recovery
	mg kg^{-1}	mg kg^{-1}	%	mg kg^{-1}	mg kg^{-1}	%	mg kg^{-1}	mg kg^{-1}	%	mg kg^{-1}	mg kg^{-1}	%
**Al**	73700	28789	39	77900	21008	27	x	7141	x	x	18765	x
**As**	10.5	12	114	23.6	22	93	3.4	3.5	103	x	15	x
**B**	74	68	92	x	11	x	x	7.6	x	x	15	x
**Ba**	x	298	x	x	56	x	304	308	101	x	78	x
**Bi**	x	<DL	x	x	0.25	x	x	<DL	x	x	0.34	x
**Ca**	19100	18965	99	x	21100	x	2600	2285	88	x	20100	x
**Cd**	0.371	0.41	111	0.73	0.85	116	0.3	0.34	113	0.67	0.61	91
**Ce**	42	37	88	x	32	x	44	37	84	56.7	51	90
**Co**	12.8	12.6	98	13.7	14.0	102	3.9	3.4	87	23	21.0	91
**Cr**	130	143	110	84	75	89	38	40	105	178	169	95
**Cu**	x	21	x	47.7	43	90	6.3	5.9	94	60	62	103
**Dy**	3	3.5	117	x	2.3	x	x	2.9	x	4.01	4.1	102
**Er**	x	1.4	x	x	2	x	x	132	x	2.35	2	89
**Eu**	0.83	0.61	73	1.25	1.20	96	0.6	0.52	87	1	1.1	110
**Fe**	33600	29459	88	37400	32655	87	9880	9553	97	44800	40890	91
**Ga**	x	1.4	x	x	5.6	x	x	2.1	x	x	4.5	x
**Gd**	3	3.10	103	x	6.7	x	x	2.8	x	4.41	4.1	93
**Ge**	x	<DL	x	x	0.23	x	x	<DL	x	x	0.04	x
**Ho**	x	0.05	x	x	0.06	x	x	0.11	x	0.8	0.71	89
**In**	x	<DL	x	x	<DL	x	x	0.23	x	x	<DL	x
**K**	21100	19724	93	24900	22287	90	12500	12199	98	x	4532	x
**La**	21.7	22	101	40.4	42	104	21	22	105	27.8	25.0	90
**Li**	x	12	x	72	66	92	x	6.5	x	x	34	x
**Lu**	0.3	0.45	150	0.468	0.54	115	0.3	0.28	93	0.325	0.39	120
**Mg**	14600	13398	92	12300	11043	90	1550	1454	94	x	4114	x
**Mn**	529	461	87	495	398	80	266	286	107	920	723	79
**Mo**	x	13	x	x	12	x	x	4	x	x	4012	x
**Na**	12200	10000	82	x	4356	x	4440	4122	93	x	5200	x
**Nd**	17	15	88	x	65	x	x	11	x	25	22	88
**Ni**	85	80	94	32.5	31	95	13	12.0	92	128	112	88
**Pb**	17.3	18	104	74.8	71	95	15	16.0	107	31.9	31	97
**Pd**	x	<DL	x	x	<DL	x	x	<DL	x	x	<DL	x
**Pr**	x	1.1	x	x	2.8	x	x	2.8	x	6.1	6.2	102
**Re**	x	0.10	x	x	0.11	x	x	0.23	x	x	0.22	x
**Rh**	x	<DL	x	x	<DL	x	x	<DL	x	x	<DL	x
**Sb**	1.55	1.8	116	1.81	1.6	88	0.50	0.46	92	0.96	0.9	93
**Sc**	11.1	10.7	96	13.52	14.0	104	4	4.1	103	13.7	14.0	102
**Se**	1.5	1.20	80	0.44	0.50	114	x	<DL	x	1.59	1.5	94
**Sm**	4	3.70	93	5.86	5.1	87	3.6	<DL	x	4.66	4.10	88
**Sr**	x	112	x	118	110	93	55	57	104	x	112	x
**Tl**	0.58	0.50	86	x	0.55	x	x	0.25	x	x	0.66	x

**Tm**	X	1.1	x	x	1.0	x	x	0.29	x	0.326	0.35	107
**Y**	2	1.9	95	x	1.8	x	x	5.9	x	x	1.8	x
**Yb**	X	1.1	x	3.04	1.1	35	2.5	2.7	108	2.2	2.1	95
**Zn**	103	98	95	279	235	84	35	38	109	175	167	95

x - no certified value

Table S3. Results of acid extractable fraction of certified reference material NIST 2709a analysis and spike recovery in standard addition method

	**NIST 2709a**	**spike**				**NIST 2709a**	**spike**		
	certified	added	determined	recovery		certified	added	determined	Recovery
	mg kg^{-1}	mg kg^{-1}	mg kg^{-1}	%		mg kg^{-1}	mg kg^{-1}	mg kg^{-1}	%
**Al**	15000	-	16300	109	**Lu**	x	5.0	4.1	82
**As**	8.2	-	7.9	96	**Mg**	10350	-	11000	106
**B**	x	5.0	4.5	90	**Mn**	415	-	388	93
**Ba**	375	-	400	107	**Mo**	x	5.0	4.0	80
**Bi**	x	5.0	5.4	108	**Na**	535	-	500	93
**Ca**	13000	-	14500	112	**Nd**	x	5.0	4.0	80
**Cd**	33.165	-	30	90	**Ni**	65	-	69	106
**Ce**	x	5.0	4.1	82	**Pb**	9.55	-	11	115
**Co**	10.6	-	12	113	**Pd**	x	5.0	4.1	82
**Cr**	56.5	-	52	92	**Pr**	x	5.0	4.6	92
**Cu**	26	-	30	115	**Re**	x	5.0	5.6	112
**Dy**	x	5.0	4.0	80	**Rh**	x	5.0	5.1	102
**Er**	x	5.0	4.0	80	**Sb**	1.35	-	8.0	81
**Eu**	x	5.0	4.0	80	**Sc**	x	5.0	4.0	80
**Fe**	24000	-	26000	108	**Se**	1.295	-	1.1	85
**Ga**	x	5.0	5.2	104	**Sm**	x	5.0	4.7	94
**Gd**	x	5.0	4.8	96	**Sr**	x	5.0	5.6	112
**Ge**	x	5.0	4.3	86	**Tl**	x	5.0	4.0	80
**Ho**	x	5.0	5.0	100	**Tm**	x	5.0	4.0	80
**In**	x	5.0	5.5	110	**Y**	x	5.0	4.9	98
**K**	3300	-	3500	106	**Yb**	x	5.0	4.1	82
**La**	x	5.0	5.8	116	**Zn**	78	-	91	86
**Li**	x	5.0	6.0	120					

x - no certified value, standard addition method used

Colorimetric analysis accuracy studies

Table S4. Spike recovery in colorimetric procedures

determined form		A		B		C	
	added	found	recovery	found	recovery	found	recovery
	mg kg^{-1}	mg kg^{-1}	%	mg kg^{-1}	%	mg kg^{-1}	%
Fe (III) using suflocyanate at 485 nm	0	9750		9839		12450	
	5000	15010	105	14820	100	16870	88
Fe (II) using 2,2'-dipirydile at 520 nm	0	319		425		720	
	250	546	91	698	109	998	111

The accuracy of measurements have been estimated by using the reference colorimetric method described previously [S4]. The flow injection analytical system has been simplified to chromatographic pump (Shimadzu, Japan), injection valve with a loop (200uL) and a diode array detector (Shimadzu, Japan) operating at two wavelengths: 396 nm and 512 nm. The reaction of both iron forms with 1,10- phenanthroline and formation of red complex with Fe(II) with maximal absorption at 512 nm and yellow complex with both forms Fe(II) and Fe(III) with equimolar absorption at 396 nm has been used. The diode array detector in flow injection system allowed to determine simultaneously contentent of Fe(II) and Fe(III) - calculated from difference Fe(III)={[Fe(II)+Fe(III)] at 396 nm}-{Fe(II) at 512 nm}

Due to the fact that the colorimetric procedure is not a fully independent reference method, the hyphenated HPLC-MIP OES and HPLC-ICP OES techniques described in the previous work [S5] have been used to determine the accuracy of the results. Microwave induced plasma optical emission spectrometry (MIP OES) and inductively coupled plasma optical emission spectrometry (ICP OES) have been used as detectors in the chromatography system (HPLC). Dionex IonPac CS5A cation exchange column and a pyridine-2,6-dicarboxylic acid (PDCA) based mobile phase have been used to separate the iron species. Optimized and validated analytical systems have been used to study the speciation of iron in a variety of samples, including samples of archaeological pottery (*Table S5*).

Table S5. Results of iron forms determination using different colorimetric procedures and hyphenated analytical systems (mg kg^{-1})

determined form	A	B	C
Fe(III) using suflocyanate at 485 nm	9750 ± 1276	9839 ± 1234	12450 ± 2076
Fe(III) using 1,10- phenanthroline at 396 nm*	9710 ± 1310	9732 ± 1198	12210 ± 2123
Fe(III) using HPLC-MIP OES	9825 ± 1638	10204 ± 1701	12560 ± 2093
Fe(III) using HPLC-ICP OES	9874 ± 1646	9751 ± 1625	12975 ± 2162
Fe(II) using 2,2'-dipirydile at 520 nm	319 ± 41	425 ±73	720 ± 112
Fe(II) using phenanthroline at 512 nm	325 ± 51	418 ± 65	730 ± 123
Fe(II) using HPLC-MIP OES	308 ± 51	413 ± 69	648 ± 108
Fe(II) using HPLC-ICP OES	311 ± 52	432 ± 72	754 ± 126

*Fe(III)={[Fe(II)+Fe(III)] at 396 nm}-{Fe(II) at 512 nm}

References to previous works

[S1] Niedzielski P., Krueger M., Brandherm D., Effects of sample processing on XRF results from archeological pottery, Materials and Manufacturing Processes 35 (2020) 1455-1460

[S2] Kozak L., Silva Souza J., Nawrot A.. Proch J., Kaźmierski M., Zawieja A., Niedzielski P., Handheld ED-XRF spectrometers in geochemical investigation – the comparative studies for glacial deposits (Spitsbergen), Polish Polar Research (2021) accepted.

[S3] Michałowski A., Niedzielski P., Kozak L., Teska M., Jakubowski K., Żółkiewski M., Archaeometrical studies of prehistoric pottery using portable ED-XRF, Measurement 159 (2020) 107758.

[S4] Kozak L., Niedzielski P., Wachowiak W., The tandem analytical method of flow injection diode array spectrophotometry and flame atomic absorption spectrometry (FI DAD(Vis)-FAAS) in iron speciation studies using 1,10-phenanthroline complexes, Microchemical Journal 110 (2013) 54-60.

[S5] Proch J., Niedzielski P., Iron species determination by high performance liquid chromatography with plasma based optical emission detectors: HPLC–MIP OES and HPLC–ICP OES, Talanta 231 (2021) 122403.
